# The magnitude of anemia and its associated factors among pregnant women in Hawela Tula Sub-city of Hawassa, Hawassa, Ethiopia

**DOI:** 10.3389/fnut.2024.1445877

**Published:** 2024-09-26

**Authors:** Sileshi Tesfaye, Legese Petros, Israel Alemayehu Tulu, Fentaw Wassie Feleke

**Affiliations:** ^1^UNICEF/LONADD, Hawassa, Ethiopia; ^2^Hosanna Health Science College, Department of Public Health, Hosanna, Ethiopia; ^3^Department of Human Nutrition, School of Human Nutrition and Food Science Technology, College of Agriculture, Hawassa University, Hawassa, Ethiopia; ^4^UNICEF, Hawassa, Ethiopia; ^5^Department of Public Health, School of Public Health, College of Medicine and Health Sciences, Woldia University, Woldia, Ethiopia

**Keywords:** anemia, ANC, Ethiopia, Hawassa, pregnant women

## Abstract

**Introduction:**

Anemia is one of the most serious health problems impacting people worldwide. The disease is quiet, moving slowly and producing only a few physical symptoms. Anemia during pregnancy raises the risk of premature birth, low birth weight, and fetal anomalies, and it can have a substantial financial impact on society and families. However, there was a paucity of studies on the magnitude and associated factors of anemia among pregnant women in southern Ethiopia.

**Objective:**

This study aimed to assess the magnitude and associated factors of anemia among pregnant women attending antenatal care in the Hawella Tula Sub-city of Hawassa City in 2021.

**Methods:**

Institution-based cross-sectional study was done on 341 randomly selected pregnant women attending antenatal care clinics. Data were obtained using a standardized semi-structured questionnaire. To identify the associated factors for the magnitude of anemia logistic regression model was used with an adjusted odds ratio (AOR) with 95% confidence intervals (CI) was calculated.

**Results:**

The prevalence of anemia among pregnant women attending antenatal care in health facilities of Hawella Tula Sub-city was 113 (33.7%) with a 95% confidence interval (CI) (28.8, 38.9). Male-headed household (AOR = 2.217, 95% CI: 1.146, 4.286), rural resident (AOR = 3.805, 95% CI: 2.118, 6.838), early marriage below 18 years (AOR = 2.137, 95% CI: 1.193, 3.830), and recurrent of illness during pregnancy (AOR = 3.189, 95% CI: 1.405, 7.241) were associated factors for anemia.

**Conclusion:**

Anemia prevalence among pregnant women was 113 (33.7%). Anemia among pregnant women was associated with rural residents, early marriage age below 18 years, and repeated illnesses during pregnancy.

## Introduction

A hemoglobin concentration below a cutoff determined by age, sex, and pregnancy is referred to as anemia (1). Low blood hemoglobin concentration, or anemia, is a health issue that affects people in high-, middle-, and low-income nations. It has detrimental implications on social and economic development and major health consequences (2). Although blood hemoglobin concentration is the most reliable indication of anemia at the population level, measures of concentration alone do not establish the cause of anemia (3). Anemia during pregnancy is classified as severe if the hemoglobin concentration is less than 7.0 g/dL, moderate if the hemoglobin concentration is between 7.0 and 9.9 g/dL, and mild if the hemoglobin concentration is between 10.0 and 11 g/dL (4).

Anemia is still a major health issue, particularly among pregnant women and girls in less developed countries (5). Anemia is thought to be a sign of both inadequate nutrition and health state (6). An estimated 2 billion people are suffering, representing more than one-third of the world’s population (7). Although nutritional, genetic, and infectious disease variables all contribute to anemia, iron deficiency accounts for 75% of cases (8, 9). Iron deficiency anemia has a detrimental impact on national development since it impairs children’s cognitive development and adult productivity (8). More than half of pregnant women and over one-third of all women suffer from anemia (10–12). In Mali, 63.5% of women of reproductive age suffer from anemia (13). In Somali land, a study found an overall prevalence of anemia among pregnant women at 50.6% (95% CI: 45.40–55.72%) (14).

In Ethiopia, the systematic review and meta-analysis pooled the prevalence of anemia among pregnant women was 26.4% (15). This is manifested by an inadequate supply of iron to the cells following the depletion of stored iron (16). Iron helps us the transfer of oxygen from the lungs to other parts of the body tissues. When iron deficiency happens it affects metabolism, brain development, and growth (17). More pregnant women are affected than other society groups (3, 18).

Anemia may result from several causes, with the most significant contributor being iron deficiency. Approximately 50% of cases of anemia are considered to be due to iron deficiency, but the proportion probably varies among population groups and in different areas, according to the local condition (19). Although the primary cause is iron deficiency, it is seldom present in isolation (20). Because of the significance of this disease worldwide, many countries have seen it cohabit more commonly with a variety of other causes, including hemoglobinopathies, dietary inadequacies, parasite infections, and malaria (21–24).

Severe maternal anemia is strongly associated with maternal mortality and is typically confounded by multiple underlying conditions. Despite this known complexity, iron deficiency is often misidentified as the single contributor to the onset of anemia. Due to their unique roles in the synthesis of hemoglobin and/or the formation of erythrocytes, deficiencies in vitamins A, B_2_, B_6_, B_12_, C, D, E, folate, copper, selenium, and zinc can also cause anemia; however, some of these micronutrients may not be significantly responsible for the prevalence of anemia worldwide, infectious diseases such as human immunodeficiency virus (HIV), parasitic infections such as hookworm and schistosomiasis and inherited genetics are additional and often neglected causes of anemia (16, 25–27).

As a result, no new research has been published in this field; however, earlier research has indicated that iron deficiency which is rarely found alone is the main contributing factor. It more often coexists with hemoglobinopathies, dietary deficits, and parasite infections. The management and control of anemia during pregnancy are significantly influenced by the availability of local prevalence information. Aside from the few studies already out in Ethiopia, nothing is currently known about the prevalence of anemia among pregnant women in our research area. To focus on the characteristics in the study region, this study aimed to determine the prevalence of anemia and its associated factors among pregnant women receiving prenatal care in Hawassa municipal administration health facilities.

## Materials and methods

### Study settings and study periods

The study was conducted at Hawela Tula sub-city of Hawassa City Administration, Sidama Regional State, Southern Ethiopia. The study was conducted from October 15 to December 2, 2021. Hawassa city was located 275 km from Addis Ababa. According to the population census, of 2007, the population of Hawassa City in 2021 was 374,034 out of which 190,757 were males and 183,277 were females (28). The total population of the sub-city is estimated to be 135,793 out of this about 4,698 (3.46%) pregnant women are expected to live in the city. In Hawassa city, there are eight sub-cities and 83 public and private health institutions among them, one public referral and teaching hospital, one General Hospital, two primary hospitals, four private hospitals, six public HC, 17 Health posts, and 51 private clinics given the service.

### Study design

A cross-sectional study design was used.

### Source and study population

All pregnant mothers living in Hawassa city were the source population. The study population was all systematically randomly selected pregnant women who had ANC follow-up in the selected health facilities.

### Inclusion and exclusion criteria

Pregnant women who had earned antenatal care at Hawella Tula Sub-city health facilities during the period under the interview were included; however, those who were seriously ill at the moment of data collection, who were unable to hear and/or speak, and who were unwilling to participate in the interviews were excluded.

### Measurement of variables

#### Outcome variable

The outcome variable in this study is anemia. According to the WHO classifications for hemoglobin concentration values, pregnant mothers were divided into four groups: Hgb level ≥ 11.0 gm/dL is no anemia, Hgb level less than 7.0 gm/dL is severe anemia, Hgb level 7–9.9 gm/dL is moderate anemia and Hgb level 10–10.9 gm/dL is mild anemia (3).

#### Independent variables

Sociodemographic and socioeconomic variables: age, religion, residence, occupational status, marital status, Educational status, monthly family income, gravidity, parity, gestational age, history of contraceptive use, history of the heavy ministerial cycle, birth interval, family size, nutritional status, hemoglobin level, intestinal parasite infestation, iron folic supplementation, iron supplementation starting period, deworming in the last 6 months, drinking of stimulants (tea, coffee, cocoa, etc.), and lack of sleeping/rest are independent variables.

Pregnant women’s nutritional status: MUAC less than 23 cm indicates that the pregnant woman has moderate malnutrition, MUAC less the 18.5 cm indicates that the mother has severe malnutrition, and MUAC 23 cm and above is normal (29).
Pregnant women DDS: Dietary Diversity Score was calculated from a single 24-h recall and all the liquids, and the foods consumed a day before the study were categorized into nine food groups. Consuming a food item from any of the groups was assigned a score of “1” and a score of “0” if the food was not taken. Thus, a Dietary Diversity Score of nine points was calculated by adding the values of all the food groups. After that, it was classified as low (less than or equal to 3) medium (4—6), and high (7—9) (30, 31).
Dietary variables: include whether tea or coffee is consumed immediately after a meal, the frequency of daily meat eating (more than once per week or less than once per week), fruit and vegetable consumption (more or less than once per day), and milk consumption.

### Sample size determination

A single population proportion is used to determine the sample size, P is obtained from similar studies in Adama town 28.1% (32). The sample size was figured out with consideration of 95% Confidence level, 5% precision, and *p* = 28.1% magnitude of anemia among pregnant women. The non-response rate taken about 10% sample in addition to the calculated “*n*.” Based on this the formula was presented as follows to calculate “*n*.”


$$\frac{n=\frac{Z\alpha }{2}*p\;\left(1–p\right)}{d^{2}}$$

Where:

*n* = the required sample size.
*p* = proportion of anemia, which is 28.1%.
*d* = estimated marginal error, which is 5%.
Z 𝛼/_2 =_ the cross-ponding value of the confidence coefficient at alpha level 0.05 which is 1.96. After adding 10%, non-response allowances the minimum final sample size became 341.

### Sampling technique and procedure

Three health institutions were selected by simple random sampling out of a total of six. Based on the family planning clinic attendants or each health center’s past performance, the proportionate sample approach was used to calculate the intended number of clients for each facility. After selecting using a lottery method from 1 to 4 for the first time, every fourth client in each health facility was interviewed to gather the necessary data for the study. This process was repeated every 4 weeks until the necessary sample size was achieved. Individual study participants were chosen using systematic sampling techniques between 1 and the sampling interval.

### Data collection tools and procedures

The semi-structured questionnaire was taken from relevant published research and then tailored to the local environment to gather critical data from expecting mothers when they exited ANC. Interviewer-assisted questions were used for socio-demographics and socioeconomic, reproductive history, maternity features, food and nutrition-related characteristics, and other variables (16, 33, 34). Variables such as hemoglobin (Hgb) level and MUAC were extracted from respondents’ charts using their registration numbers. Dietary diversity was assessed using the 24-h dietary recall approach, which was modified from the Food and Agriculture Organization criteria for quantifying household and individual dietary diversity (30, 31). The selected interviewers employed questionnaires and interview procedures to obtain data from expectant mothers at the selected health facilities. The data collection method was undertaken by a team of four senior midwives and two experienced supervisors.

### Data quality control

To ensure the accuracy of the results, a mid-upper arm circumference measurement device and a 10% pretested semi-structured questionnaire were utilized. Three days of training on the purpose of the study, how to contact study participants, and how to utilize the questionnaire were given to the data collectors and supervisors. Senior experts with extensive experience performed the laboratory procedures and sample collection. The gathered data were checked for accuracy, clarity, and completeness by the principal investigator and supervisors. Subsequent data collection, this quality check was conducted daily, and any modifications were made before the subsequent data collection measure. The data were cleaned up and cross-checked before analysis. The outcome and independent variables were coded before putting the data into software to ensure data completeness and consistency. The data were then entered into Epi-data version 4.6.2 software and exported to SPSS version 25 software for data analysis.

### Data analysis

Demographic information and other factors were presented using descriptive statistics such as frequency, and percentage. Furthermore, data were illustrated with tables, graphs, and texts. The data were analyzed using both bivariate and multivariable logistic regression models to identify associations. In the bivariate analysis, factors having a *p* value less than 0.25 were chosen for further examination in the multivariable logistic model. To ensure model fitness, the multi-collinearity and goodness of Hosmer and Lemeshow tests were applied. This multivariable approach aims to find significant variables while controlling for any confounding factors. Adjusted Odds Ratios (AORs) were used to measure relationships, along with a 95% confidence interval. Variables having a *p* value equal to or less than 0.05 were considered statistically significant, indicating a strong association or influence in the context of the research.

## Results

### Socio-demographic characteristics of the respondents

A total of 335 pregnant mothers among pregnant women attending antenatal care in Hawella Tula Sub-city of Hawassa city administration with a response rate of 98.22% took part in the study. The mean (±SD) age was 27.4 (±4.3) years, which categorized as mainly 188 (56.1%) were 25–29 years. Most of the respondents 246 (73.4%) were headed by males and 234 (69.9%) were urban dwellers. About the educational level of the mothers, 79 (23.6%) had no formal education and 39 (11.6%) of them attended college and above education. About half of the study participants 176 (52.5%) were housewives. About the husband’s educational status, 40 (11.9%) had no formal education. The mean (±SD) family size was 4.2 (±1.9), and the age at first marriage for 112 (33.4%) was below the age of 18 years (Table 1).

**Table 1:** Socio-demographic characters of pregnant women attending antenatal care in Hawella Tula Sub-city in Hawassa City, Ethiopia, 2021.

Variables (*n* = 335)	Category	No (%)
Age (years)	15–24	71 (21.2)
	25–29	188 (56.1)
	30–34	55 (16.4)
	35 and above	21 (6.3)
Sex of household head	Female	89 (26.6)
	Male	246 (73.4)
Place of residence	Rural	101 (30.1)
	Urban	234 (69.9)
Mother’s education	No formal education	79 (23.6)
	Primary	132 (39.4)
	Secondary	85 (25.4)
	College diploma and above	39 (11.6)
Husband’s education	No formal education	40 (11.9)
	Primary	116 (34.6)
	Secondary	105 (31.3)
	College diploma and above	74 (22.1)
Family size	≤2	76 (22.7)
	3–4	142 (42.4)
	≥5	117 (34.9)
Size of farmland	0.5 ha	189 (56.4)
	0.5–1 ha	106 (31.6)
	> 1 ha	40 (11.9)
Age of first marriage	< 18 years	112 (33.4)
	≥ 18 years	223 (66.6)
Occupation	Housewife	176 (52.5)
	government employed	24 (7.2)
	Private employee	50 (14.9)
	Private business	85 (25.4)
Profession of mothers	Health Science	107 (31.9)
	Education	80 (23.8)
	Business and social science	18 (5.4)
	Non health natural science	130 (38.8)
Husband’s profession	Health Science	16 (4.8)
	Education	51 (15.2)
	Agriculture	40 (11.9)
	Business and social science	16 (4.8)
	Non health natural science	212 (63.3)
Monthly income	< 25 USD $	225 (67.2)
	25–42 USD $	90 (26.9)
	> 42 USD $	20 (6.0)

### Obstetric characteristics of study participants

The number of pregnancies with a mean (±SD) was 3.1 (±1.4) and the majority of the study participants 277 (82.7%) were multi-gravida, 231 (69.0%) were part 2–4, and most of the reports 334 (99.7%) had single pregnancy. About half of the respondents 186 (48.2%) were between 1 and 3 months of gestational age on their first visit and 70 (20.9%) only had a history of abortion. Out of the study participants interviewed, 134 (47.5%) had below 24 months birth interval and 263 (78.5%) had regular iron intake. On dietary habits, 193 (57.6%) had meat consumption and 146 (43.6%) were using tea immediately after having a meal (Table 2).

**Table 2:** Individual-related factors of pregnant women attending antenatal care in Hawella Tula Sub-city in Hawassa City, Ethiopia, 2021.

Variables (*n* = 335)	Category	No.(%)
Gravidity	Primigravidae	58 (17.3)
	Multigravida	277 (82.7)
History of abortion	No	265 (79.1)
	Yes	70 (20.9)
Parity	Nullipara	60 (17.9)
	One	23 (6.9)
	Two to four	231 (69.0)
	≥ five	21 (6.3)
Regular antenatal checkups	Yes	263 (78.5)
	No	72 (21.5)
Birth interval	≥ 24 months	134 (47.5)
	< 24 months	148 (52.5)
Regular intake of iron	Yes	263 (78.5)
	No	72 (21.5)
Meat consumption	Yes	193 (57.6)
	No	142 (42.4)
Intake of tea after meal	Yes	146 (43.6)
	No	189 (56.4)
Recurrent illness during pregnancy	No	297 (88.7)
	Yes	38 (11.3)
No of children	1–3	147 (53.5)
	4–6	126 (45.8)
	Above 7	2 (0.7)
Type of pregnancy	Single	334 (99.7)
	Twin	1 (0.3)
Rate of flow of menstruation	Very heavy	84 (25.1)
	Heavy	69 (20.6)
	Moderate	125 (37.3)
	Low	57 (17.0)

### Health and nutrition service-related factors

Many of the study participants 243 (72.5%) travel less than 30 min to reach the nearest health institution. Nearly half of the study participants, 160 (47.8%) had nutritional counseling. Most of the participants 218 (65.1%) had experience with food fortification and 285 (85.1%) used piped water for drinking. Furthermore, 275 (82.1%) had latrines and 108 (32.2%) had deworming in the last six mothers during pregnancy preparation (Table 3).

**Table 3:** Health and nutrition-related service of pregnant women attending antenatal care in Hawella Tula Sub-city in Hawassa City, Ethiopia, 2021.

Variables (*n* = 335)	Category	No (%)
Nutritional counseling	No	175 (52.2)
	Yes	160 (47.8)
Distance from home to health institution	< 30 min	243 (72.5)
	≥30 min	92 (27.5)
Food fortification	Yes	218 (65.1)
	No	117 (34.9)
Iron supplementation starting time	1–3 months	162 (48.4)
	4–5 months	55 (16.4)
	6–7 months	67 (20.0)
	8–9 months	51 (15.2)
Source of drinking water	Pipe water	285 (85.1)
	Springs	28 (8.4)
	Wells	22 (6.6)
Availability of latrine	Yes	275 (82.1)
	No	60 (17.9)
Deworming in last 6 month	Yes	108 (32.2)
	No	227 (67.8)
DDS	Low	236 (70.4)
	Medium	60 (17.9)
	High	39 (11.7)
MUAC of mother	<23 cm	72 (21.5)
	≥23 cm	263 (78.5)
Meal frequency	<3	61 (18.2)
	≥3	274 (81.8)

### The magnitude of anemia in pregnant women

**Figure 1:**
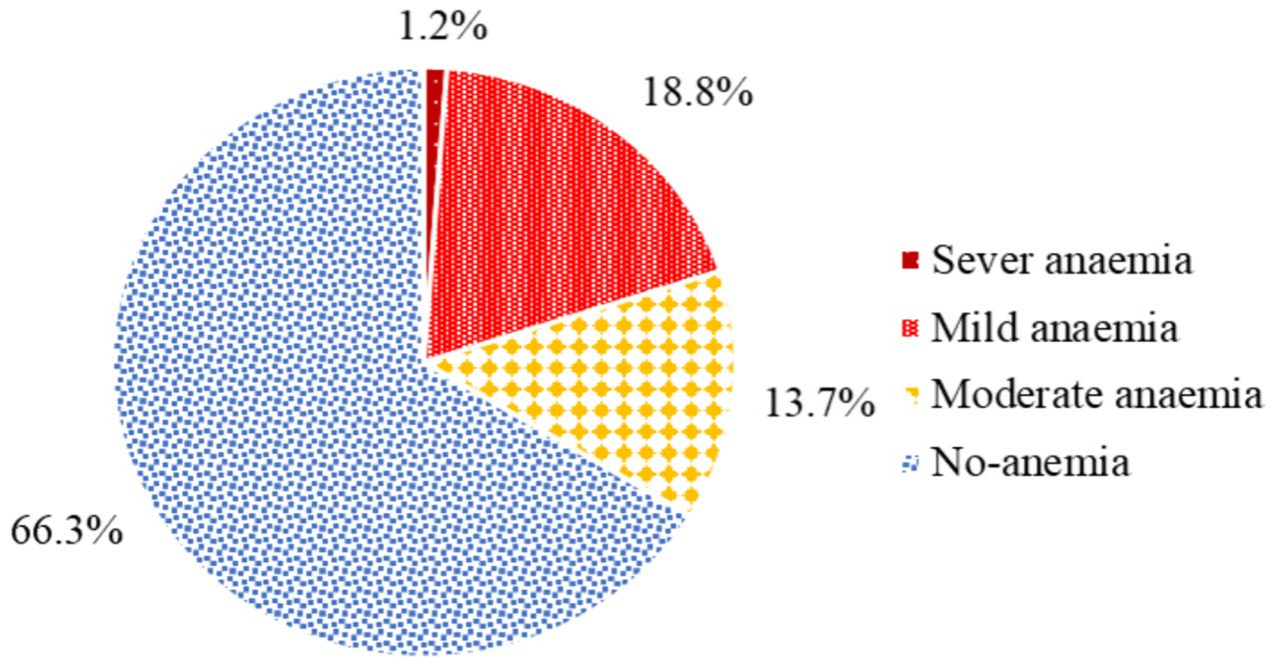
The magnitude of anemia among pregnant women attending antenatal care in Hawella Tula Sub-city in Hawassa City, Ethiopia, 2021.

The magnitude of anemia among pregnant women attending antenatal care in health facilities of Hawella Tula Sub-city was 113 (33.7%) with 95% CI (28.8, 38.9). Among these, 4 (1.2%) had severe anemia, 63 (18.8%) had mild anemia, and 46 (13.7%) had moderate anemia (Figure 1).

### Factors associated with the prevalence of anemia

The maternal meal frequency, maternal DDS, maternal MUAC, and iron folic supplementation, were not candidate variables for the prevalence of anemia. The sex of the head of household, place of residence, age at first marriage, birth interval, rate of menstrual flow, history of abortion, recurrence of illness during pregnancy, and nutritional counseling were found to be candidate variables for multivariate analysis and be associated with the prevalence of anemia, with *p*-values <0.25. In the multivariable logistic regression, the pregnant women from male-headed households were about 2.2 times more likely to develop anemia (AOR = 2.217, 95% CI: 1.146, 4.286) as compared with female-headed households. In addition to this rural resident were about 3.8 times more likely to develop anemia (AOR = 3.805, 95% CI: 2.118, 6.838) as compared with urban dwellers. Early marriage below 18 years (AOR = 2.137, 95% CI: 1.193, 3.830) and recurrent illness during pregnancy (AOR = 3.189, 95% CI: 1.405, 7.241) were associated factors for the prevalence of anemia as compared with their counterparts (Table 4).

**Table 4:** Bivariable and multivariable logistic regression analysis model for prevalence of anemia among pregnant women in Hawella Tula Sub-city in Hawassa city, Southern Ethiopia, 2021.

Variables (*n* = 335)		Anemia		COR (95%CI)	AOR (95%CI)	*p* value
		Yes	No			
		No. (%)	No. (%)			
Sex head of household						
	Male	95 (38.6)	151 (61.4)	2.482(1.393, 4.421)	2.217 (1.146, 4.286)	0.018*
	Female	18 (20.2)	71 (79.8)	1	1	
Place of residence						
	Rural	52 (51.5)	49 (48.5)	3.010 (1.849, 4.899)	3.805 (2.118, 6.838)	<0.001**
	Urban	61 (26.1)	173 (73.9)	1	1	
Age of first marriage						
	< 18 years	50 (44.6)	62 (55.4)	2.048 (1.276, 3.287)	2.137 (1.193, 3.830)	0.011*
	≥ 18 years	63 (28.3)	160 (71.7)	1	1	
Birth interval						
	< 24 months	59 (39.9)	89 (60.1)	1.615 (0.982, 2.655)	1.548 (0.885, 2.708)	0.126
	≥ 24 months	39 (29.1)	95 (70.9)	1	1	
Rate of flow of menstruation						
	Heavy	59 (38.8)	93 (61.2)	2.307 (1.098, 4.849)	2.009 (0.856, 4.713)	0.109
	Moderate	43 (32.6)	89 (67.4)	1.757 (0.822, 3.757)	1.280 (0.534, 3.070)	0.580
	Low	11 (21.6)	40 (78.4)	1	1	
History of abortion						
	Yes	33 (47.1)	37 (52.9)	2.062 (1.205, 3.531)	1.648 (0.876, 3.100)	0.121
	No	80 (30.2)	185 (69.8)	1	1	
Recurrent illness during pregnancy						
	Yes	21 (55.3)	17 (44.7)	2.753 (1.387, 5.461)	3.189 (1.405, 7.241)	0.006*
	No	92 (31.0)	205 (69.0)	1	1	

COR, Crude odds ratio; AOR, Adjusted odd ratio; *p value < 0.05, **p value < 0.001.

## Discussion

Anemia prevalence was reported to be 33.7% among pregnant women receiving prenatal care at health facilities. It was almost similar to research (36.6%) carried out in western Arsi (35), Ilu Aba Bora zone in southwest Ethiopia (31.5%) (33), and Arba Minch Town in southern Ethiopia (32.8%) (36), and eastern Africa (34.85%) (37). But higher other study findings were conducted in western Ethiopia (23.5%) (38), Southeast Ethiopia (27.9%) (39), Addis Ababa, Ethiopia (21.3%) (40), Azezo health center, Ethiopia (21.1%) (41) and in Gondar town (16.6%) (42). It was also lower in the study conducted in India (84.85%) (43), Nepal (48%) (44), Libya (54.6%) (45), Kenya (57%) (46), Africa (42.17%) (47), and at Bahirdar Felege-Hiwot Referral Hospital Northwestern Ethiopia (45.5%) (48). The observed discrepancy could be explained by differences in sociodemographic factors and geographical variance among research participants, which could be explained by the occurrence of food taboos during pregnancy in specific Ethiopian communities. For example, according to a study conducted in Tigray (12%) (49), and in Addis Ababa (18.2%) (50) women skipped at least one food category during their recent pregnancy. Furthermore, this variation might be attributed to the difference in methods and lifestyle between study participants (51).

Compared to female-headed households, male-headed households are linked to anemia. This outcome was consistent with the Pakistani study (52). Nevertheless, this contradicts data from eastern Africa that was obtained using a multilevel mixed-effects generalized linear model (37). One possible explanation for this discrepancy could be the mother’s workload when she enters the workforce; in particular, the amount of time spent on time-consuming home tasks like child care and breastfeeding tends to decrease (53, 54).

This study demonstrated that there is a significant association between rural resident pregnant women and anemia. Compared to urban residents, those who live in rural areas are more susceptible to anemia. This outcome was in line with the research conducted in rural Sidama (55), and Arba Minch Town, Southern Ethiopia (36), Tigray (56), and Australia (57). The higher risk of anemia among women from rural areas who come to hospitals for labor may be related to a lack of information about adequate nutrition during pregnancy, economic factors, diet differences, or the inaccessibility of healthcare services (40). To reduce severe anemia, women need to be empowered economically, play a great role in decision-making, and receive counseling services for dietary diversification (51). Poor sanitation conditions in rural areas can result in inadequate restrooms and sources of drinking water, as evidenced by consistent reports from the Lao People’s Democratic Republic (27).

This study showed that early marriage below 18 years was associated with anemia. This result was in line with the study in Bahir Dar Felege-Hiwot Referral Hospital Northwestern Ethiopia (48), India (43), and Nepal (44). This is because early marriages below 18 years might be exposed to undernutrition due to maternal demand and pregnancy (47). This could be because adolescence is a period of increased iron demands due to the growth of muscle mass and blood volume in addition to sharing with the developing fetus. Due to menstrual blood loss, young women are more vulnerable to the development of iron deficiency. This evidence has been confirmed by Ethiopian data supporting the 23.8% overall prevalence of anemia among female adolescents showed a substantial correlation between early marriage and childbearing and teenage anemia (58).

There was a strong significant association between recurrent illness during pregnancy and anemia among pregnant women. Those mothers who were infected by recurrent illness were 3.189 times more likely to develop anemia than those who were not recurrently ill during the current pregnancy. This was similar to the study result in western Ethiopia (38), and in Arba Minch Town in southern Ethiopia (36). This could be because of blood loss caused by parasite infestations, which could put women at risk of iron deficiency anemia. Studies from Durame, south Ethiopia, reinforce this evidence (56), Dessie and Yirgalem (59, 60). This may also be linked to the likelihood that environmental contamination of drinking water with parasites that cause anemia increases due to the lack of accessible restrooms, which may raise the risk of anemia (27, 61). This might be happening due to the impacts of lead poisoning in the workplace and environment related to iron metabolism (62).

The nutrition-related factors of maternal DDS, meal frequency, MUAC, and iron-folic acid supplementation during pregnancy are significantly associated variables in this study result. For example, pregnant women who did not take iron supplements during their pregnancy were more likely to develop anemia than those who did. This could be associated with an iron deficiency that occurs during pregnancy as a result of increased iron requirements to supply the mother’s growing blood volume as well as the rapidly growing fetus and placenta. The findings are comparable with studies conducted in Karnataka, India, Uganda, and Ethiopia (63–66).

Pregnant women with a low dietary variety score were more likely to develop anemia than those with an optimal dietary diversity score. This finding was validated by research conducted in Mekele Town, Hosan, and South Ethiopia (34, 67, 68). Inadequate dietary diversification can result in a shortage of micronutrients such as vitamins, minerals, and other necessary substances, which can boost iron bioavailability and modify iron levels. This could be because pregnancy requires biologically high nutrition; it is recommended to adjust one’s diet from the norm.

### Study strengths and limitations

Despite this study’s attempt to provide accurate information about the study objective and confidentiality during the interview, there is a chance of recall and social desirability bias in the participants’ number of pregnancies, abortion history, and socioeconomic status. Furthermore, we used the WHO-recommended technique for developing countries, and Hgb levels were not detected using the gold standard method.

## Conclusion

The magnitude of anemia among pregnant women was found 33.7%. Factors such as male head of the family, living in a rural area, being married young (before turning 18), and experiencing repeated illnesses while pregnant were found to be associated factors. Emphasis should be placed on gender roles, the proper age for marriage, and nutrition education regarding the consumption of foods high in iron during prenatal care, especially in rural communities.

## Data Availability

The raw data supporting the conclusions of this article will be made available by the authors, without undue reservation.

## References

[1] World Health Organization (2011). Haemoglobin concentrations for the diagnosis of anaemia and assessment of severity. Vitamin and mineral nutrition information system. Document reference WHO. NMH/NHD/MNM/11.1. Geneva, Switzerland. Available online at: http://www.who.int/entity/vmnis/indicators/haemoglobin (Accessed September 15, 2021).

[2] Stevens G Finucane MM de-Regil LM Paciorek CJ Flaxman SR Branca F. Global, regional, and national trends in haemoglobin concentration and prevalence of total and severe anaemia in children and pregnant and non-pregnant women for 1995–2011: a systematic analysis of population-representative data. Lancet Glob Health. (2013) 1:e16–25. doi: 10.1016/S2214-109X(13)70001-9, PMID: 25103581 PMC4547326

[3] World Health Organization (WHO) (2017). Progress in partnership: 2017 progress report on the every woman every child global strategy for women’s, children’s and adolescents’ health.

[4] WHO (2011). Haemoglobin concentrations for the diagnosis of Anaemia and assessment of severity, Geneva. WHO.

[5] Safiri S Kolahi AA Noori M Nejadghaderi SA Karamzad N Bragazzi NL. Burden of anemia and its underlying causes in 204 countries and territories, 1990–2019: results from the global burden of disease study 2019. J Hematol Oncol. (2021) 14:1–16. doi: 10.1186/s13045-021-01202-2 34736513 PMC8567696

[6] McLean E Cogswell M Egli I Wojdyla D de Benoist B. Worldwide prevalence of anaemia, WHO vitamin and mineral nutrition information system, 1993–2005. Public Health Nutr. (2009) 12:444–54. doi: 10.1017/S1368980008002401 18498676

[7] World Health Organization (2001). Prevention and control of iron-deficiency anaemia in women and children: Report of the UNICEF.

[8] Balarajan Y Ramakrishnan U Özaltin E Shankar AH Subramanian SV. Anaemia in low-income and middle-income countries. Lancet. (2011) 378:2123–35. doi: 10.1016/S0140-6736(10)62304-5 21813172

[9] Salhan S Tripathi V Singh R Gaikwad HS. Evaluation of hematological parameters in partial exchange and packed cell transfusion in treatment of severe anemia in pregnancy. Anemia. (2012) 2012:608658. doi: 10.1155/2012/608658 22693662 PMC3368167

[10] Al-Aini S Senan CP Azzani M. Prevalence and associated factors of anemia among pregnant women in Sana’a, Yemen. Indian J Med Sci. (2020) 72:185–90. doi: 10.25259/IJMS_5_2020

[11] Ogunbode O Ogunbode O. (2021). Anaemia in Pregnancy. In: Okonofua F Balogun JA Odunsi K Chilaka VN Editors. Contemporary Obstetrics and Gynecology for Developing Countries. Springer: Cham. 321–330. doi: 10.1007/978-3-030-75385-6_29

[12] Helion Belay AM Tariku A Woreta SA Demissie GD Asrade G. Anemia and associated factors among pregnant women attending prenatal Care in Rural Dembia District, north West Ethiopia: a cross-sectional study. Ecol Food Nutr. (2020) 59:154–74. doi: 10.1080/03670244.2019.1680551 31657236

[13] Armah-Ansah EK. Determinants of anemia among women of childbearing age: analysis of the 2018 Mali demographic and health survey. Arch Public Health. (2023) 81:10. doi: 10.1186/s13690-023-01023-4, PMID: 36658651 PMC9854152

[14] Abdilahi MM Kiruja J Farah BO Abdirahman FM Mohamed AI Mohamed J. Prevalence of anemia and associated factors among pregnant women at Hargeisa group hospital, Somaliland. BMC Pregnancy Childbirth. (2024) 24:332. doi: 10.1186/s12884-024-06539-3, PMID: 38724919 PMC11080199

[15] Geta TG Gebremedhin S Omigbodun AO. Prevalence and predictors of anemia among pregnant women in Ethiopia: systematic review and meta-analysis. PLoS One. (2022) 17:e0267005. doi: 10.1371/journal.pone.0267005, PMID: 35895619 PMC9328503

[16] Stephen G Mgongo M Hussein Hashim T Katanga J Stray-Pedersen B Msuya SE. Anemia in pregnancy: prevalence, risk factors, and adverse perinatal outcomes in northern Tanzania. Hindawi Publish Corp. (2018) 2018:1–9. doi: 10.1155/2018/1846280 PMC5954959 29854446

[17] Casanueva E Viteri FE. Iron and oxidative stress in pregnancy. J Nutr. (2003) 133:1700S–8S. doi: 10.1093/jn/133.5.1700S 12730487

[18] Mekonnen FA Ambaw YA Neri GT. Socio-economic determinants of anemia in pregnancy in north Shoa zone, Ethiopia. PLoS One. (2018) 13:e0202734. doi: 10.1371/journal.pone.0202734, PMID: 30133527 PMC6105028

[19] Kaushal S Priya T Thakur S Marwaha P Kaur H. The etiology of Anemia among pregnant women in the hill state of Himachal Pradesh in North India: a cross-sectional study. Cureus. (2022) 14:e21444. doi: 10.7759/cureus.21444, PMID: 35223229 PMC8857868

[20] World Health Organization (2012). Daily iron and folic acid supplementation in pregnant women. 23586119

[21] Mavondo GA Mzingwane ML. Severe malarial anemia (SMA) pathophysiology and the use of phytotherapeutics as treatment options. Curr Top Anem. (2017) 20:189–214. doi: 10.5772/intechopen.70411

[22] Gasim GI Adam I. Malaria, schistosomiasis, and related anemia In: Nutritional Deficiency, Pınar E. Belma K.-G. Editors. Rijeka. Ch. 7: IntechOpen. (2016) doi: 10.5772/63396

[23] White NJ. Anaemia and malaria. Malar J. (2018) 17:1–17. doi: 10.1186/s12936-018-2509-9 30340592 PMC6194647

[24] Hess SY Owais A Jefferds MED Young MF Cahill A Rogers LM. Accelerating action to reduce anemia: review of causes and risk factors and related data needs. Ann N Y Acad Sci. (2023) 1523:11–23. doi: 10.1111/nyas.14985, PMID: 36987993 PMC10918744

[25] Green R. Datta Mitra A. (2010). Anemia Resulting From Other Nutritional Deficiencies. William’s Hematology. New York, NY: McGraw-Hill, 607–612

[26] Chaparro CM Suchdev PS. Anemia epidemiology, pathophysiology, and etiology in low-and middle-income countries. Ann N Y Acad Sci. (2019) 1450:15–31. doi: 10.1111/nyas.14092, PMID: 31008520 PMC6697587

[27] Keokenchanh S Kounnavong S Tokinobu A Midorikawa K Ikeda W Morita A. Prevalence of anemia and its associate factors among women of reproductive age in Lao PDR: evidence from a nationally representative survey. Anemia. (2021) 2021:1–9. doi: 10.1155/2021/8823030, PMID: 33520310 PMC7822650

[28] Lenjiso T Tesfaye B Michael A Esatu T Legessie Z. (2013). SNNPR Hawassa city Adminstration health department 2010–2012 GTP assessment report booklet. p. 9–65.

[29] Ethiopia Ministry of Health (2019). Management of Severe Acute Malnutrition: Ethiopia-federal ministry of health, Addis Ababa.

[30] Deriba BS Bulto GA Bala ET. Nutritional-related predictors of anemia among pregnant women attending antenatal care in Central Ethiopia: an unmatched case-control study. Biomed Res Int. (2020) 2020:1–9. doi: 10.1155/2020/8824291 PMC7691012 33294455

[31] Kennedy G. Ballard T. Dop M. (2011). Guidelines for measuring household and individual dietary diversity.

[32] Mohammed E Mannekulih E Abdo M. Magnitude of anemia and associated factors among pregnant women visiting public health institutions for antenatal care services in Adama town, Ethiopia. Centr Afric J Public Health. (2018) 4:149–58. doi: 10.11648/j.cajph.20180405.14

[33] Kenea A Negash E Bacha L Wakgari N. Magnitude of anemia and associated factors among pregnant women attending antenatal care in public hospitals of ilu Abba bora zone, south West Ethiopia: a cross-sectional study. Anemia. (2018) 2018:1–7. doi: 10.1155/2018/9201383, PMID: 30538862 PMC6257898

[34] Delil R Tamiru D Zinab B. Dietary diversity and its association with anemia among pregnant women attending public health facilities in South Ethiopia. Ethiop J Health Sci. (2018) 28. doi: 10.4314/ejhs.v28i5.14 PMC6308774 30607078

[35] Niguse O Andualem M Teshome G. Magnitude of Anemia and associated risk factors among pregnant women attending antenatal care in Shalla Woreda, West Arsi Zone, Oromia Region, Ethiopia. Ethiop J Health Sci. (2013) 23:165–73. 23950633 PMC3742894

[36] Alemayehu B Marelign T Aleme M. Prevalence of Anemia and its associated factors among pregnant women attending antenatal Care in Health Institutions of Arba Minch town, Gamo Gofa zone, Ethiopia. Hindawi Publish Corp. (2016) 2016:1–9. doi: 10.1155/2016/1073192 PMC4779815 27022481

[37] Teshale AB Tesema GA Worku MG Yeshaw Y Tessema ZT. Anemia and its associated factors among women of reproductive age in eastern Africa: a multilevel mixed-effects generalized linear model. PLoS One. (2020) 15:e0238957. doi: 10.1371/journal.pone.0238957, PMID: 32915880 PMC7485848

[38] Zekarias B Meleko A Hayder A Nigatu A Yetagessu T. Prevalence of Anemia and its associated factors among pregnant women attending antenatal care (ANC) in Mizan Tepi university teaching hospital, south West Ethiopia. Health Sci J. (2017) 11:559. doi: 10.21767/1791-809X.1000529

[39] Kefiyalew F Zemene E Asres Y Gedefaw L. Anemia among pregnant women in Southeast Ethiopia: prevalence, severity and associated risk factors. BMC Res Notes. (2014) 7:771. doi: 10.1186/1756-0500-7-771 25362931 PMC4223834

[40] Jufa A Zewde T. Prevalence of anemia among pregnant women attending antenatal care at Tikur Anbessa specialized hospital, Addis Ababa Ethiopia. J Hematol Thromb Dis. (2014) 2:125. doi: 10.4172/2329-8790.1000125

[41] Alem M Enawgaw B Gelaw A Kenaw T Seid M Olkeba Y. Prevalence of anemia and associated risk factors among pregnant women attending antenatal care in Azezo health center Gondar town, Northwest Ethiopia. J Interdiscip Hist. (2013) 1:137–44. doi: 10.5455/jihp.20130122042052

[42] Melku M Addis Z Alem M Enawgaw B. Prevalence and predictors of maternal anemia during pregnancy in Gondar, Northwest Ethiopia: an institutional based cross-sectional study. Anemia. (2014) 2014:108593:1–9. doi: 10.1155/2014/108593 24669317 PMC3942101

[43] Khan NAM Sonkar VRK Domple VK Inamdar IA. Study of Anaemia and Its Associated Risk Factors among Pregnant Women in a Rural Field Practice Area of a Medical College. Natl J Community Med [Internet]. (2017). 8:396–400. Available at: https://www.njcmindia.com/index.php/file/article/view/1110

[44] Rayamajhi N. 16. Prevalence of Anemia in pregnant women attending a tertiary level Hospital in Western Region, Nepal. J Univ Coll Med Sci. (2016) 4:17–9.

[45] Raga AE Mariam O. Prevalence of anaemia among pregnant women in Derna city, Libya. Int J Commun Med Public Health. (2016) 3:1915–20.

[46] Okube OT Mirie W Odhiambo E Sabina W Habtu M. Prevalence and factors associated with Anaemia among pregnant women attending antenatal Clinic in the Second and Third Trimesters at Pumwani maternity hospital, Kenya. Open J Obstet Gynecol. (2016) 6:16–27. doi: 10.4236/ojog.2016.61003

[47] Worku MG Alamneh TS Teshale AB Yeshaw Y Alem AZ Ayalew HG. Multilevel analysis of determinants of anemia among young women (15-24) in sub-Sahara Africa. PLoS One. (2022) 17:e0268129. doi: 10.1371/journal.pone.0268129, PMID: 35533194 PMC9084531

[48] Mulugeta M Aster A. Maternal anemia during pregnancy in Bahirdar town, northwestern Ethiopia: a facility-based retrospective study. Appl Med Res. (2015) 11:2–7. doi: 10.5455/amr.20150129110510

[49] Tela FG Gebremariam LW Beyene SA. Food taboos and related misperceptions during pregnancy in Mekelle city, Tigray, northern Ethiopia. PLoS One. (2020) 15:e0239451. doi: 10.1371/journal.pone.0239451, PMID: 33048926 PMC7553351

[50] Mohammed SH Taye H Larijani B Esmaillzadeh A. Food taboo among pregnant Ethiopian women: magnitude, drivers, and association with anemia. Nutr J. (2019) 18:19. doi: 10.1186/s12937-019-0444-4, PMID: 30904017 PMC6431010

[51] Agbozo F Abubakari A der J Jahn A. Maternal dietary intakes, red blood cell indices and risk for anemia in the first, second and third trimesters of pregnancy and at predelivery. Nutrients. (2020) 12:777. doi: 10.3390/nu12030777, PMID: 32183478 PMC7146471

[52] Khalid S Hafeez A Mashhadi SF. Frequency of Anemia in pregnancy and its association with socio-demographic factors in women visiting a tertiary care hospital in Rawalpindi: Anemia in Pregnancy. Pak Armed Forces Med J. (2017) 67:19–24.

[53] Marcus H Schauer C Zlotkin S. Effect of Anemia on work productivity in both labor-and nonlabor-intensive occupations: a systematic narrative synthesis. Food Nutr Bull. (2021) 42:289–308. doi: 10.1177/03795721211006658, PMID: 33874760

[54] Abbi R Christian P Gujral S Gopaldas T. The impact of maternal work status on the nutrition and health status of children. Food Nutr Bull. (1991) 13:1–6. doi: 10.1177/156482659101300128

[55] Gebremedhin S Enquselassie F Umeta M. Prevalence and correlates of maternal anemia in rural Sidama, southern Ethiopia. Afr J Reprod Health. (2014) 18:44–53. PMID: 24796168

[56] Weldekidan F Kote M Girma M Boti N Gultie T. Determinants of anemia among pregnant women attending antenatal clinic in public health facilities at durame town: unmatched case control study. Anemia. (2018) 2018:1–8. doi: 10.1155/2018/8938307 PMC6174810 30345112

[57] Hildingsson I Haines H Cross M Pallant JF Rubertsson C. Women's satisfaction with antenatal care: comparing women in Sweden and Australia. Women Birth. (2013) 26:e9–e14. doi: 10.1016/j.wombi.2012.06.002, PMID: 22795867

[58] Tiruneh FN Tenagashaw MW Asres DT Cherie HA. Associations of early marriage and early childbearing with anemia among adolescent girls in Ethiopia: a multilevel analysis of nationwide survey. Arch Public Health. (2021) 79:91. doi: 10.1186/s13690-021-00610-7, PMID: 34082813 PMC8173845

[59] Argaw BA Argaw-Denboba A Taye B Worku A Worku A. Major risk factors predicting anemia development during pregnancy: unmatched-case control study. J Commun Med Health Educ. (2015) 5:2161–0711. doi: 10.4172/2161-0711.1000353

[60] Tadesse SE Seid O G/Mariam Y Fekadu A Wasihun Y Endris K. Determinants of anemia among pregnant mothers attending antenatal care in Dessie town health facilities, northern Central Ethiopia, unmatched case-control study. PLoS One. (2017) 12:e0173173. doi: 10.1371/journal.pone.0173173, PMID: 28288159 PMC5348124

[61] Ngure FM Reid BM Humphrey JH Mbuya MN Pelto G Stoltzfus RJ. Water, sanitation, and hygiene (WASH), environmental enteropathy, nutrition, and early child development: making the links. Ann N Y Acad Sci. (2014) 1308:118–28. doi: 10.1111/nyas.12330, PMID: 24571214

[62] Słota M Wąsik M Stołtny T Machoń-Grecka A Kasperczyk S. Effects of environmental and occupational lead toxicity and its association with iron metabolism. Toxicol Appl Pharmacol. (2022) 434:115794. doi: 10.1016/j.taap.2021.115794, PMID: 34780723

[63] Gebreweld A Tsegaye A. Prevalence and factors associated with anemia among pregnant women attending antenatal clinic at St. Paul’s hospital millennium medical college, Addis Ababa, Ethiopia. Adv Hematol. (2018) 2018:3942301. doi: 10.1155/2018/3942301 30245724 PMC6136568

[64] Addis Alene K Mohamed Dohe A. Prevalence of anemia and associated factors among pregnant women in an urban area of eastern Ethiopia. Anemia. (2014) 2014:561567. doi: 10.1155/2014/561567 25215230 PMC4158560

[65] Viveki RG Halappanavar AB Viveki PR Halki SB Maled VS Deshpande PS. Prevalence of anaemia and its epidemiological determinants in pregnant women. Al Ameen J Med Sci. (2012) 5:216–23.

[66] Mbule MA Byaruhanga YB Kabahenda M Lubowa A. Determinants of anaemia among pregnant women in rural Uganda. Rural Remote Health. (2013) 13:1–15. doi: 10.22605/RRH2259 23679828

[67] Abriha A Yesuf ME Wassie MM. Prevalence and associated factors of anemia among pregnant women of Mekelle town: a cross sectional study. BMC Res Notes. (2014) 7:1–6. doi: 10.1186/1756-0500-7-888 25487251 PMC4295569

[68] Teshome MS Meskel DH Wondafrash B. Determinants of anemia among pregnant women attending antenatal care clinic at public health facilities in Kacha Birra District, southern Ethiopia. J Multidiscip Healthc. (2020) 13:1007–15. doi: 10.2147/JMDH.S259882, PMID: 33061406 PMC7522419

